# Case report: A case of radiation-induced diffuse hemorrhagic gastroduodenitis successfully treated with thalidomide

**DOI:** 10.3389/fphar.2026.1809335

**Published:** 2026-07-31

**Authors:** Yifan Zhang, Liming Zhang, Feng Zhang, Yulan Liu, Ning Chen

**Affiliations:** 1 Department of Gastroenterology, Peking University People’s Hospital, Beijing, China; 2 Clinical Center of Immune-Mediated Digestive Diseases, Peking University People’s Hospital, Beijing, China; 3 Endoscopy Center, Peking University People’s Hospital, Beijing, China

**Keywords:** gastroduodenitis, refractory hemorrhage, radiotherapy complication, thalidomide, case report

## Abstract

Radiation-induced hemorrhagic gastroduodenitis is an intractable and potentially life-threatening condition. This study reports the case of a 57-year-old male with radiation-induced hemorrhagic gastroduodenitis. The patient had previously received radiotherapy for metastatic tumors involving the liver and adrenal gland. Following this treatment, he presented with melena and anemia, necessitating hospital admission. Prior therapeutic efforts included epinephrine spraying in the gastric antrum and embolization of the gastroduodenal artery. Despite these measures, along with multiple transfusions of packed red blood cells, the hemorrhage persisted. Ultimately, oral administration of thalidomide successfully controlled the bleeding. This case indicates that oral thalidomide may represent a potential treatment option for severe radiation-induced hemorrhagic gastroduodenitis.

## Introduction

Radiation-induced hemorrhagic gastritis or gastroduodenitis represents a severe and clinically challenging complication (Zhang et al., 2012; Tatsis et al., 2016). Despite the availability of multiple therapeutic approaches, a consensus gold-standard management strategy remains undefined. In this study, we report a case of intractable gastroduodenal hemorrhage following postoperative radiotherapy that was successfully managed with oral thalidomide.

## Case description

**FIGURE 1:**
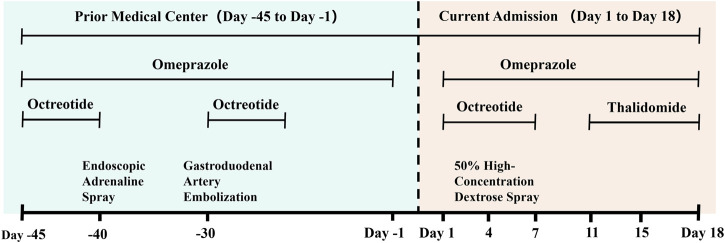
Schematic representation of the patient’s clinical management timeline. Prior interventions (Day −45 to Day −1) included octreotide, omeprazole, endoscopic adrenaline spray, and gastroduodenal artery embolization. During the current admission (Day 1 to Day 18), octreotide, omeprazole, 50% dextrose spray, and thalidomide (started Day 11) were administered.

A 57-year-old male patient with primary renal and bladder tumors developed abdominal distension, melena, and hematemesis during radiotherapy and was admitted to our hospital. After the gastric tube was inserted, it was drained, revealing dark red contents from the stomach. He previously underwent a right nephrectomy and kidney transplantation approximately 10 years ago. Reexamination showed multiple liver metastases and left adrenal gland metastases 1 year ago. He received radiotherapy for a liver metastatic tumor (45 Gy in 15 times) and an adrenal gland metastatic tumor (90 Gy in 30 times) for approximately 3 months. He was treated with endoscopic adrenaline spray and gastroduodenal artery interventional embolization, which were ineffective in controlling the active bleeding (Figure 1). Laboratory tests performed at an external hospital indicated moderate anemia, with a hemoglobin level of 56 g/L.

**FIGURE 2:**
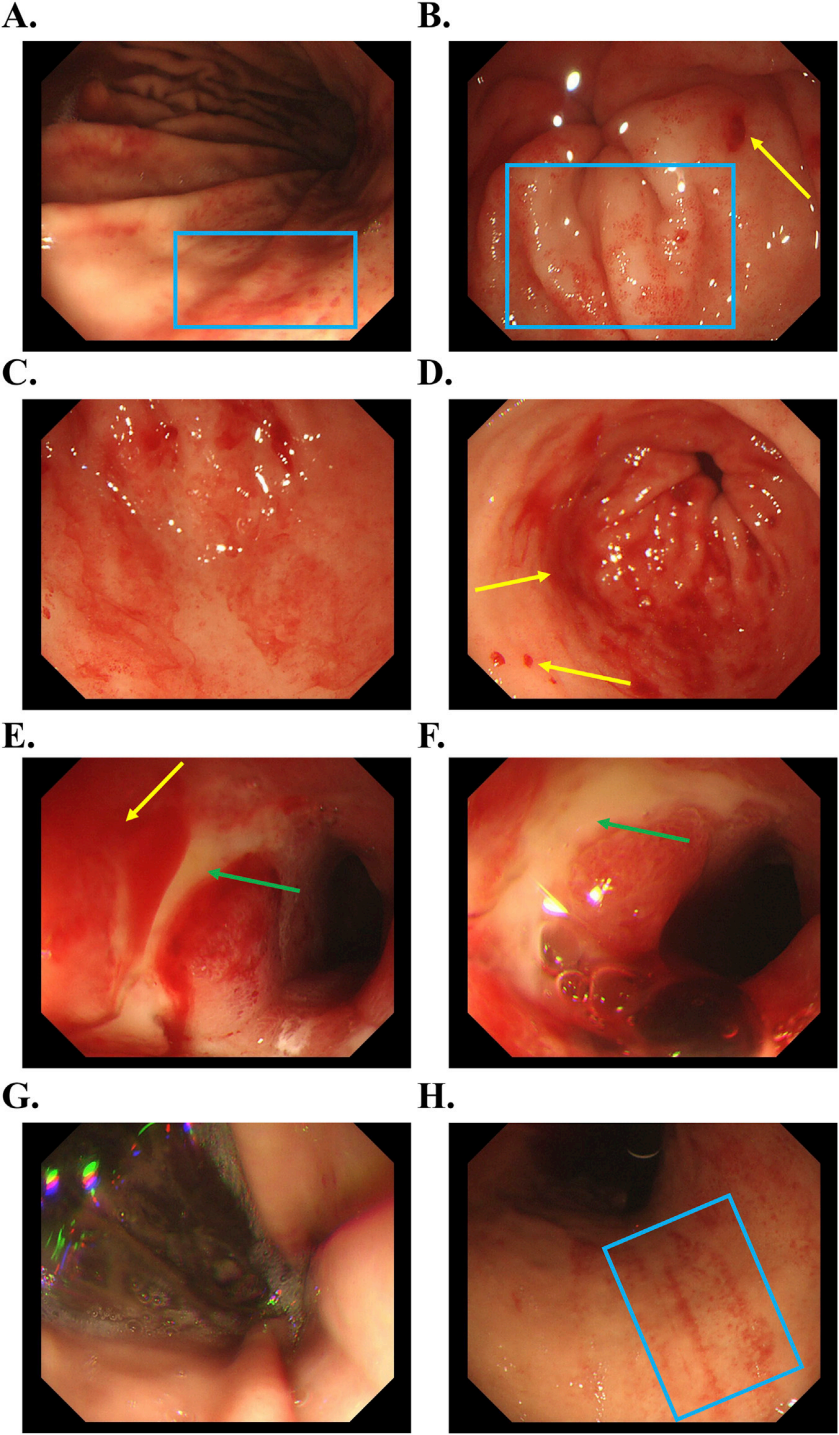
**(A–H)** Representative endoscopic images showing radiation-induced gastroduodenitis. **(A)** Gastric body; **(B)** gastric antrum; **(C)** gastric antrum (after rinsing); **(D)**. gastric antrum (few seconds after rinsing); **(E)** duodenum of descending and transition; **(F)** duodenum of descending and transition (after rinsing); **(G)** mucus pool; **(H)** gastric body. Blue boxes indicate typical telangiectasia; yellow arrows indicate active oozing; and green arrows indicate duodenal ulcers.

**FIGURE 3:**
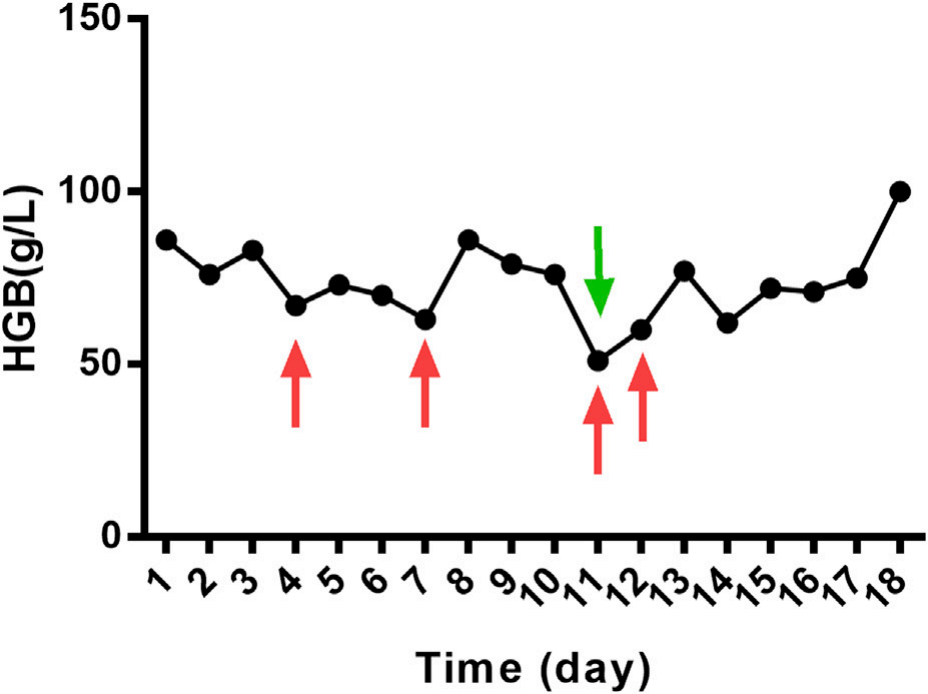
Hemoglobin trends during the current hospitalization. Red arrows indicate blood transfusion; the green arrow indicates the initiation of thalidomide treatment.

During his hospitalization at our institution, management included octreotide acetate (25 μg/h by continuous intravenous infusion), omeprazole sodium (40 mg intravenously every 12 hours), and packed red blood cell transfusions (patient details are summarized in Table 1; Figure 1). Nevertheless, intermittent upper gastrointestinal bleeding persisted, with a notable progression of melena. Subsequent endoscopic evaluation revealed diffuse gastric mucosal edema, hyperemia, and telangiectasia throughout the entire stomach. Additionally, multiple hemorrhagic patches with active oozing were observed over the antrum. A duodenal ulcer with active oozing (Forrest Ib) was noted at the junction of the descending duodenum (Figure 2). He was diagnosed with radiation-induced gastroduodenitis based on the findings of previous examinations. Initial endoscopic application of 50% high-concentration dextrose spray to the gastric mucosa was attempted. However, this approach failed to adequately control the extensive hemorrhagic lesions. Oral thalidomide therapy was subsequently administered. Within 2 weeks of initiating thalidomide, hemorrhage was controlled, with resolution of both melena and hematemesis. The patient’s stool normalized from melena to yellow, soft consistency, and hemoglobin levels recovered to 100 g/L (Figure 3). During a 6-month post-discharge follow-up, the patient had no recurrence of hematemesis or melena. However, the patient ultimately succumbed to progressive systemic metastasis.

**TABLE 1:** Baseline laboratory findings.

Information	Sex	Male
	Year	57
Blood routine examination	WBC	4.78 × 10^9^/L
	RBC	2.62 × 10^9^/L
	Hb	86 g/L
	HCT	27.3%
	PLT	83 × 10^9^/L
	CRP	49.7 mg/L
	ESR	41 mm/h
Biochemical test	ALT	14 U/L
	AST	15 U/L
	Tbil	13.3 µmol/L
	Cr	72 µmol/L
	eGFR	98.43 mL/min*1.73 m^2^
	ALB	36.8 g/L
	BNP	59 pg/mL
	Mb	22.6 ng/mL
	Tn	<2.0 pg/mL
	CK-MB	0.7 ng/mL
	PT	12.7 s
	APTT	83 s
	INR	1.12
	FDP	8.6 µg/mL
	D-dimer	988 ng/mL
	K	3.61 mmol/L
	Na	146.4 mmol/L
	Cl	113.9 mmol/L
	TG	1.68 mmol/L
	TC	3.51 mmol/L
	HDL	1.04 mmol/L
	LDL	1.92 mmol/L
	AFP	14.14 ng/mL
	CEA	0.8 ng/mL

## Discussion

In recent years, radiotherapy has been recognized as a viable therapeutic approach for liver metastasis in cases where surgical resection is not feasible, especially when combined with other modalities such as intra-arterial chemotherapy (Zhang et al., 2012; Tatsis et al., 2016). However, radiation-induced gastritis stands out as the most severe and therapeutically challenging complication. The underlying reason lies in the pronounced radiosensitivity of gastric tissue, which has a tolerated dose limit of approximately 45 Gy. Consequently, patients undergoing radiotherapy for liver metastasis are at a heightened risk of developing gastroduodenal toxicity. Risk factors for gastric injury are primarily associated with high daily frequency and elevated cumulative radiation dose (Wada et al., 2003). The early stage typically presents as acute gastric mucosal inflammation. If the injury progresses, vascular pathology may worsen, characterized by progressive obliterative endarteritis and endothelial proliferation, which results in mucosal ischemia, ulcer formation, and the development of telangiectasias (Wada et al., 2003; Shukuwa et al., 2007). The clinical presentation of radiation gastritis commonly includes symptoms such as abdominal pain, distension, loss of appetite, nausea, and vomiting. Endoscopic or imaging findings may further reveal mucosal ulcers and luminal stenosis. In more severe instances, the condition can progress to upper gastrointestinal hemorrhage (Afifi et al., 2020). Duodenal ulceration superimposed on radiation-induced gastritis is relatively uncommon. Notably, the failure of this ulcer to demonstrate any healing tendency despite nearly 6 weeks of standardized acid-suppressive therapy, in conjunction with its location in the descending duodenum—an atypical site for peptic ulceration—strongly suggests that the underlying pathogenetic mechanism is not primarily driven by acid-mediated erosion, but rather is more consistent with the pathological characteristics of radiation-induced injury. Radiation-induced gastritis or gastroduodenitis can lead to intractable bleeding characterized by a diffuse pattern with hemorrhage originating from multiple sites. Conventional management strategies, including antisecretory agents and transfusion therapy, are often inadequate for achieving effective hemostasis. The condition typically presents with clinical manifestations such as melena and hematemesis, which commonly emerge 2–3 months after the initiation of radiotherapy (Toyoda et al., 2004). Endoscopic examination typically reveals characteristic findings, including mucosal telangiectasias, edema and diffuse erythema. Additional observations may comprise shallow or deep ulcerations and areas of scarring.

Previous studies have shown that endoscopic argon plasma coagulation (APC) (Liang et al., 2018; Ross et al., 2012), subtotal gastrectomy (Tatsis et al., 2016) and prednisolone (Zhang et al., 2012; Craanen et al., 2006) may be effective treatment options for radiation-induced hemorrhagic gastritis. As illustrated by this case history, due to the advanced tumor burden, interventions such as gastrectomy or prednisolone therapy are often challenging. Studies have established that hypertonic glucose spray can effectively control active bleeding in radiation enteritis with diffuse mucosal hemorrhage (Tian et al., 2014). Nonetheless, for this patient, treatment using a 50% high-concentration dextrose spray failed to achieve sustained hemostasis.

To date, there are no prior reports describing the use of thalidomide in managing radiation-induced gastritis or gastroduodenitis. It is noteworthy that thalidomide possesses dual mechanisms of action, exerting anti-inflammatory effects while also functioning as a potent angiogenesis inhibitor, primarily via the suppression of vascular endothelial growth factor (VEGF). In addition to its anti-inflammatory properties, thalidomide also acts as a potent angiogenesis inhibitor. This pharmacological profile suggests its potential utility in managing intestinal bleeding that is refractory to conventional therapies, including conditions such as radiation-induced proctitis (Craanen et al., 2006; Bauditz et al., 2004; Alberto et al., 2008). The selection of a 100 mg daily dose of thalidomide in this case was guided by a balance between demonstrated antiangiogenic efficacy and safety considerations specific to this patient’s clinical context. Although the patient was 57 years old, he exhibited an overall fragile status characterized by end-stage metastatic disease with multi-organ involvement and recent high-dose radiotherapy, factors that substantially compromise physiological reserve and drug tolerability. Evidence from randomized controlled trials of thalidomide for vascular gastrointestinal bleeding supports 100 mg daily as an effective dose, with response rates of 71.4% (Ge et al., 2011) and 68.6% (Chen et al., 2023), the latter trial further demonstrating that even 50 mg daily achieves clinically meaningful bleeding reduction (51.0% response rate). Notably, concurrent radiotherapy has been specifically associated with heightened thalidomide toxicity (Anscher et al., 2006; Hoang et al., 2012). In light of the patient’s palliative condition, post-radiotherapy status, and the principle of minimizing iatrogenic risk in end-stage malignancy, a 100 mg daily dose was deemed judicious and appropriate.

It is crucial to acknowledge a major limitation of the present report. Owing to the patient’s end-stage tumor condition and palliative care status, a follow-up gastroscopy was not conducted to visually assess the mucosal response to thalidomide therapy. Although the cessation of melena and hematemesis, together with the recovery of hemoglobin levels, provides clinically meaningful evidence for the control of active hemorrhage, these parameters alone do not confirm anatomical healing at the mucosal level. Clinical bleeding cessation does not necessarily imply the regression of telangiectasias, resolution of mucosal edema, or re-epithelialization of ulcerative lesions. Given the absence of such visual confirmation in the present case, the favorable outcome reported herein should be interpreted cautiously as clinical hemostasis rather than endoscopically confirmed mucosal healing. Nevertheless, given the significant risks posed by recurrent hemorrhage and the favorable safety profile of thalidomide (Vasiliauskas et al., 1999; Ehrenpreis et al., 1999), our findings suggest that it represents a viable therapeutic alternative for cases of radiation-induced gastritis that are refractory to standard therapeutic approaches.

## Conclusion

To the best of our knowledge, the application of thalidomide for managing radiation-induced gastroduodenitis has not been previously documented. The present case illustrates a potential novel therapeutic strategy for this condition. In summary, thalidomide may serve as an effective treatment alternative for patients presenting with refractory radiation gastroduodenitis.

## Data Availability

The raw data supporting the conclusions of this article will be made available by the authors, without undue reservation.
